# To the Editors of the Medical and Physical Journal

**Published:** 1806-07

**Authors:** W. Clement

**Affiliations:** Shrewsbury


### *To the Editors of the Medical and Physical Journal.*

GENTLEMEN,

THE Friends of Humanity will rejoice at the favourable Report given by Dr. Wood, of Newcastle, in his "History of the Progress and Success of Vaccination" at that place, published in No. 84 of your Journal. In some remarks, (Journal 75) I had no intention of *accusing* the Doctor of any crime, by his not producing in a former Report "those sterling instructions as well as observations given by Dr. Jenner on that particular point;" but I was certainly then of opinion, and have since been confirmed, by his subsequent Report, that he did not believe in, or had not observed *herpes* to disturb or prevent perfect vaccination.

In stating those observations on Dr. Jenner's excellent paper, I had no object in view but to serve the cause of truth; and no doubt Dr. W. is influenced by the same motive, however widely we may differ in opinion. On other particular points of his paper, I have been fully and ably anticipated by Mr. Creaser, of Bath, Journal 86. I am now warranted to affirm, from my own experience, (which has not been inconsiderable, having the sole care of the poor of six united parishes, attached to the Shrewsbury House of Industry) that herpetic, and other irritative eruptions, in far the greatest number of cases, induce varieties in the vaccine pustule, inflammation, and scabbing.

The definition of a spurious pustule is well given by Mr. Creaser, and, I presume, the test of perfect vaccination is the regular, undisturbed progress of the vesicle, inflammation, and subsequent scabbing. In few cases, indeed, are we able to detect any constitutional affection. I concur with Dr. Wood in recommending both arms to be inoculated. We have thereby more certainty of infection taking place, and we have likewise a greater resource of vaccine fluid; but I cannot subscribe to the principle, that the constitution is better secured by the *quantum* of morbid matter to be absorbed. From September last to January I inoculated between two and three hundred, in which number were included *four herpetic cases*, and one with itch. *One* only out of the four had the regular appearance; the itch patient was likewise incompleat, owing to the pustule having been scratched.

 October

October the 24th, 1805, I inoculated with fresh fluid, Thomas Evans, aged 35 years, William Evans, five, and Sarah Evans, two years old. The father and daughter passed through the disease regularly; William had been herpetic for a twelvemonth before, his arm appeared very forward on the third day; the fifth and sixth, the pustule increased towards prematurity, inflammation deeper coloured, and the vesicle at last yielded a light, soft, honeycomb scab. The eruptions about the body did not in the least partake of the vaccine influence. I again inoculated him on the seventh of this month, which took effect, and *he has passed through the disease as regularly as any patient I ever saw.* He was at the time of his last inoculation, and is now, perfectly free from any cuticular eruption. "After the *system* has been *vaccinated,* a second inoculation with the vaccine matter will not produce a pustule similar in its stages, or its appearance, to that produced by the first inoculation." In these sentiments, I cordially agree with Dr. Wood, by only placing my last inoculation in the above case, where the first ought to have been. Thus, then, Dr. Jenner's position stands beyond a possibility of doubt, that "vaccination on an herpetic skin produces every gradation in the state of the pustule, from that slight deviation from peefection, which is quite immaterial, up to that point which affords no security at all.

I am, &c.

*Shrewsbury, May* 22, 1806. W. CLEMENT.

---

